# Aflibercept in clinical practice; visual acuity, injection numbers and adherence to treatment, for diabetic macular oedema in 21 UK hospitals over 3 years

**DOI:** 10.1038/s41433-021-01625-8

**Published:** 2021-07-09

**Authors:** S. J. Talks, I. Stratton, T. Peto, A. Lotery, U. Chakravarthy, H. Eleftheriadis, S. Izadi, N. Dhingra, P. Scanlon, the UK Aflibercept Users’ Group, James Talks, Peter Scanlon, Quresh Mohamed, Andrew Lotery, Sharam Kashani, Nasos Georgas, Colin Jones, Abdisattar Gashut, Cynthia Santiago, Romi Chhabra, Richard Antcliff, Naren Dhingra, Clare Bailey, Usha Chakravarthy, Tunde Peto, Faruque Ghanchi, Linda Mcinerney, Salim Natha, Rehna Khan, Indra Dias, Raj Mukhrejee, Shahrnaz Izadi, Irfan Tahir, Haralabos Eleftheriadis

**Affiliations:** 1 The Newcastle Upon Tyne hospitals NHS Foundation Trust, Newcastle Upon Tyne, UK; 2 Gloucestershire Hospitals NHS Foundation Trust, Gloucester, UK; 3 Belfast Health and Social Care Trust, Belfast, UK; 4 Faculty of Medicine, University of Southampton, Southampton, UK; 5 Kings College Hospital NHS Foundation Trust, London, UK; 6 Epsom and St Helier University Hospitals NHS, Epsom, UK; 7 The Mid Yorkshire Hospitals NHS Trust, Yorkshire, UK; 8 University Hospital Southampton NHST, Gloucester, UK; 9 East Sussex Hospitals NHS FT, Saint Leonards-on-sea, UK; 10 Hinchingbrooke Health Care NHS Trust, Huntingdon, UK; 11 Norfolk and Norwich University Hospitals NHS Foundation Trust, Norwich, UK; 12 Wirral University Teaching Hospital NHS Foundation Trust, Wirral, UK; 13 NHS Grampian, Aberdeen, UK; 14 Central Manchester University Hospitals NHS FT, Manchester, UK; 15 Royal United Hospitals Bath NHS Foundation Trust, Avon, UK; 16 University Hospitals Bristol NHS Foundation Trust, Bristol, UK; 17 Bradford Teaching Hospitals NHS Foundation Trust & Airedale NHS Foundation Trust, Bradford, UK; 18 Peterborough and Stamford Hospitals NHS Foundation Trust, Peterborough, UK; 19 Wrightington, Wigan and Leigh NHS Foundation Trust, Wigan, UK; 20 Calderdale and Huddersfield NHS Foundation Trust, Huddersfield, UK; 21 Leeds Teaching Hospitals NHS Trust, Leeds, UK; 22 Royal Berkshire NHS Foundation Trust Prince Charles Eye Unit, Windsor, UK

**Keywords:** Eye diseases, Scientific community

## Abstract

**Introduction:**

Randomised controlled trials provide evidence that a treatment works. Real world evidence is required to assess if proven treatments are effective in practice.

**Method:**

Retrospective data collection on patients given aflibercept for diabetic macular oedema over 3 years from 21 UK hospitals: visual acuity (VA); Index of multiple deprivation score (IMD); injection numbers; protocols used, compared as a cohort and between sites.

**Results:**

Complete data: 1742 patients (from 2196 eligible) at 1 year, 860 (from 1270) at 2, 305 (from 506) at 3 years. The median VA improved from 65 to 71, 70, 70 (ETDRS letters) at 1, 2 and 3 years with 6, 9 and 12 injections, respectively. Loss to follow-up: 10% 1 year, 28.8% at 3. Centres varied: baseline: mean age 61–71 years (*p* < 0.0001); mean IMD score 15–37 (*p* < 0.0001); mean VA 49–68 (*p* < 0.0001). Only four centres provided a loading course of five injections at monthly intervals and one 6. This did not alter VA outcome at 1 year. Higher IMD was associated with younger age (*p* = 0.0023) and worse VA at baseline (*p* < 0.0001) not total number of injections or change in VA. Lower starting VA, higher IMD and older age were associated with lower adherence (*p* = 0.0010).

**Conclusions:**

The data showed significant variation between treatment centres for starting age, VA and IMD which influenced adherence and chances of good VA. Once treatment was started IMD did not alter likelihood of improvement. Loading dose intensity did not alter outcome at one year.

## Introduction

Randomised controlled trials provide evidence of the efficacy of a treatment. Real world evidence (RWE) assesses the effectiveness of a treatment when introduced into routine clinical practice [1]. For anti-vascular endothelial growth factor (anti-VEGF) treatment, RWE can be used for measuring outcomes such as numbers of injections given over time and adherence to follow-up. In addition, RWE can be used to compare centres for patient population characteristics and treatment provision in order to provide evidence enabling service improvements [2]. On the contrary, comparing VA outcomes for different treatment regimes, using multi-centre data collection might not be as robust due to influences such as service capacity, clinician choice, patient adherence and payer requirements.

The VIVID and VISTA studies showed that treatment with aflibercept, an anti-VEGF, in a clinical setting resulted in a mean visual acuity (VA) gain of 10 ETDRS (Early Treatment Diabetic Retinopathy Study) letters, from a mean baseline of 60 letters, at 1 year, in eyes with DME [3]. This was similar to that seen in the RISE and RIDE studies for ranibizumab [4]. In the VIVID and VISTA trials, five injections were given a month apart then 2 monthly for a year, forming the basis of aflibercept’s posology in DME. In Protocol T of DRCR.net, initially four injections were given 4 weeks apart supplemented by two more injections 4 weeks apart if DME was still present [5], leading to some clinicians promoting a protocol of 6 injections at 4 weekly intervals [5]. Previously published RWE in the UK for ranibizumab found that, when compared to VIVID and VISTA, the mean starting VA was lower at 51.2 letters and VA gains modest, increasing to 54.2 and 52.5 letters at 1 and 2 years respectively, but an average of only 3.3 injections were actually given during the first year [6].

Ranibizumab was approved as a first-line treatment in the UK for centre involving DME over 400 microns in 2012 and aflibercept followed in 2015. Aflibercept has replaced ranibizumab as first-line DME treatment in many centres. The aim of this study was to assess the effectiveness of aflibercept in improving and preserving vision in patients with DME in clinical practice over three years in the UK.

We assessed the number of injections given, initial treatment protocols used, changes in VA and adherence to treatment in a multicentre cohort of patients and specifically examined differences between centres. To assess potential differences between patient populations in participating centres we correlated this with the index of multiple deprivation score (IMD), mean age, mean duration of diabetes, proportion of diabetes type, and mean baseline VA. In addition, we used survival analysis as a means of assessing outcomes which is less affected by annual time points and loss to follow-up [7].

## Methods

Relevant anonymised data of DME patients undergoing aflibercept injections from 21 UK hospitals that used a dedicated ophthalmology electronic patient record system (Medisoft Ophthalmology, Medisoft Limited, Leeds, UK) was exported in December 2019. If both eyes were treated, only data from the first eye to be treated were included; for those in whom treatment was commenced simultaneously in both eyes, the eye with better VA was used. Cases were excluded from analysis If additional pharmaceutical therapy had been given. All data from patients who had a baseline VA measurement and the potential for 1, 2 or 3 years of follow-up was accounted for. Included in the 1, 2 and 3-year VA outcome analysis were those who had, both baseline VA and a VA measurement within 8 weeks of the specified year point and a further measurement beyond that year point.

The lead clinician and Caldicott Guardian (responsible nominee for data protection) at each NHS hospital gave written approval for anonymised data extraction. Anonymized database analyses of this type do not require ethical permission as they are viewed as service evaluation. This study was conducted in accordance with the Declaration of Helsinki 2013 and the United Kingdom’s Data Protection Act 2018.

VA records are entered as ETDRS letter scores at 2 m in the EMR system. At each visit, the best-measured VA value was used in the analysis, usually with a habitual correction rather than refraction, as this is routine clinical practice. VA of count fingers, hand movements, perception of light (PL) and no PL were substituted with a value of 0 letters. Both the mean and median values are reported in this study as the data are unlikely to be normally distributed, and the median is less influenced by outliers, such as very low VA in patients with vitreous haemorrhage.

Linear regression was used to relate VA change over 12 months from the first injection with age (grouped <65 years, 65–74, 75 and above), baseline letter score (<50 letters, 50–59, 60–69, 70–79, 80 or more), and number of injections. The Cox proportional hazards model was used for survival analysis of time to VA improvement in relation to injection numbers and time to lost to follow-up.

The number of patients completing an initial course of 3, 5 and 6 monthly injections was analysed and total number of injections per treatment regime used was compared between centres.

In the UK deprivation is measured using the IMD [8]. The relationship between IMD and the age of presentation, VA, number of injections given and adherence to follow-up was assessed.

All statistical analyses were carried out using SAS 9.4. Descriptive statistics for baseline characteristics were obtained using PROC MEANS and PROC FREQ and plots done with PROC GPLOT. Time to event data was analysed using PROC LIFETEST and PROC PHREG.

## Results

In total 2196 patients at the 21 sites had an aflibercept injection for DME at least 1 year prior to data extraction and had a baseline VA measurement, being eligible for 1-year follow-up. Of these, 226 had no data at or beyond 1 year (10%). An additional 228 had no data within 8 weeks of the anniversary. Altogether 1742 were analysed for the 1-year outcomes. Corresponding numbers for year 2 and 3 are given in Table 1.

**Table 1:** Numbers of patients with potential data and actual data over three years.

	Year 1	Year 2	Year 3
Total number of patients	2196	1270	506
Excluded from annual time point: no data within 8 weeks but has data at a further time point	228	163	58
No data at or beyond annual time point	226 (10%)	241 (19.5%)	146 (28.8%)
Total analysed for annual time points	1742	860	305

There was no significant heterogeneity between centres in duration of diabetes (mean 15–19 years, *p* = 0.68). The majority had type 2 diabetes (86%) and 62% were male (Table 2).

**Table 2:** Demographics and visual acuity (VA) changes at 1, 2 and 3 years.

	One-year cohort	Two-year cohort	Three-year cohort
*N*	1742	860	305
Age at first injection (years) (Median 25th to 75th centile)	64 (56–73)	64 (56–72)	63 (55–72)
Gender (M/F)	61%/39 %	62%/38%	63%/37%
Type of diabetes (T1DM/T2DM)	14%/86%	14%/86%	15%/85%
Time since diagnosis of diabetes (years)	15 (10–21)	15 (9–21)	14 (8–21)
Injections (*n*)	6 (5–8)	9 (6–11)	12 (7–16)
VA at start of period (letters) (Median ETDRS letters)	64 (55–74)	65 (54–72)	65 (55–72)
VA at anniversary (letters) (Median EDTRS letters)	71 (60–78)	70 (60–78)	70 (60–77)
VA gain (letters)	5 (−1 to 12)	5 (−2 to 12)	5 (−2 to 13)

There was significant heterogeneity between centres for baseline age (61–71 (*p* < 0.0001)), IMD score (mean 15–37 (*p* < 0.0001), and VA (49–68 letters *p* < 0.0001). Deaths were recorded in 119 cases, varying from 0% to 7.5% between centres.

The 1742 patients in the 1-year cohort had a median age of 64 years (56–73 years; 25th to 75th centile), mean baseline VA 62 (ETDRS) letters (median 65; (Inter Quartile Range IQR 55 to 74), improving by 5 letters (IQR-1 to 12) to a mean of 67, median of 71 (IQR 60–78), after a median of 6 (IQR 5 to 8) injections. Worse baseline VA was associated with the greatest improvement in VA (*p* < 0.001), those with fewer than 50 letters at baseline having improved 13.9 letters (19.3 mean standard deviation (SD)) and those with 80 letters or more losing 3.2 (7.0 SD) letters.

The 860 patients with 2-year data had a median baseline VA of 65 letters (IQR 54-72) and median VA at 2 years of 70 (IQR 60–78) after a median of 9 (6–11) injections.

For the 305 patients with 3 years of follow-up, the median VA at baseline was 65 (IQR 55 to 72) and at 3 years 70 (IQR 60–77), after a median of 12 (IQR 7–16) injections. The median OCT thickness reduced from 441(IQR 394–506) to 283 μm at 3 years, (IQR 241 to 316).

Older patients had a smaller VA improvement (*p* = 0.0002), >75 years and above gaining 4.2 (14.1 SD) letters and <55 years gaining 6.6 (14.2 SD) letters. After adjustment for age group and baseline VA each additional injection gave an improvement of 0.56(0.14) letters (*p* < 0.0001).

An alternative parameterisation of the data uses survival analysis to look at time to sustained gain of ten letters, using number of injections as a time-dependent co-variate in a Cox proportional hazards model. This model shows that those with better VA and those who are older are less likely to gain ten letters and also that at each time point having had one more injection increased the probability of achieving a ten-letter gain, hazard ratio 1.10 (95% CI 1.06–1.15).

**Fig. 1:**
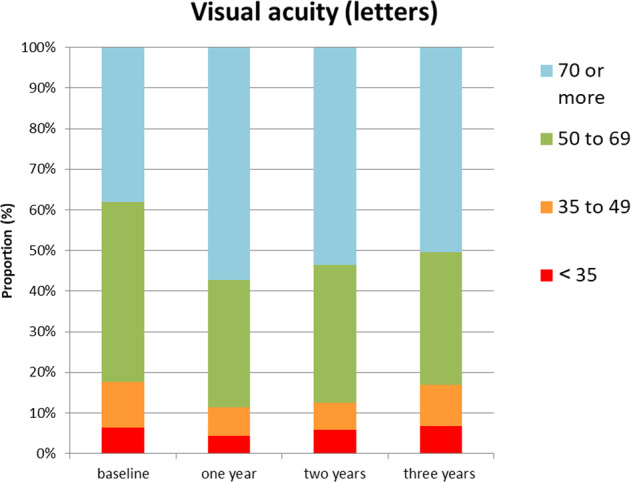
ETDRS letter score colour bands at each year time point. Proportion of patients, who had data, with different levels of visual acuity (ETDRS letter scores) at 1, 2 and 3 years.

The proportion of patients with different levels of vision is given in Fig. 1. Patients with >70 letters increased from 39% at baseline to 56% at 1 year, 55% at 2 and 51% at 3 years, for those still under follow-up.

We analysed which sites used a 3, 5 or 6 initial monthly injection protocol and whether that influenced the total number of injections given over time or the VA outcomes. (Table 3) A three-injection loading dose protocol defined as giving three injections 28 days apart was given in 1393/1742 (80%), with substantial difference between centres (35–100%). Altogether 256 patients had a five-injection loading protocol, the majority in four centres who completed this in 75–79% of cases. Only 86 patients received a six injection loading phase, the majority of whom were from one centre, which used this protocol in 80% of their patients.

**Table 3:** Proportion of Patients given three, five or six injection initial treatment protocols in the different centres and the relation to visual acuity (VA) over 1 year.

Variable	*N* (3)	Mean (s.d.)	Median (25th to 75th centile)	*N* (5)	Mean (s.d.)	Median (25th to 75th centile)	*N* (6)	Mean (s.d.)	Median (25th to 75th centile)	*P* value anova	*P* value non param
Age at first injection	1393	63.9 (12.8)	64 (56–73)	256	63.5 (13.2)	64 (55– 72.5)	86	63.5 (13.2)	65 (56–76)	0.7	0.6
years since diagnosis of diabetes	838	16.4 (10.2)	15 (10–21)	147	16 (10.3)	15 (9–20)	50	16 (10.3)	14.5 (6– 19)	0.9	0.6
VA (letters) at first injection	1393	61.3 (16.1)	65 (54–73)	256	64.8 (14.4)	68 (58–75)	86	64.8 (14.4)	70 (55–75)	0.003	<0.001
VA (letters) at one year	1393	66.7 (15.9)	70 (60–78)	256	71.3 (14.1)	75 (67.5– 80)	86	71.3 (14.1)	70 (56–79)	<0.001	<0.001
Gain (letters)	1393	5.4 (13.4)	5 (−1 to 11)	256	6.5 (13.2)	6 (0–12)	86	6.5 (13.2)	4 (−5 to 12)	0.076	0.10
Number of injections in first year	1393	5.9 (2)	6 (5–7)	256	6.8 (1.9)	7 (5–8)	86	6.8 (1.9)	7 (6–7)	<0.001	<0.001

*N* number of patients with data available; (3)(5)(6) protocol used.

We found no difference in the VA at 1 year regardless of the initial treatment protocol used and the average number of injections was the same, although there was an association with more improvement with more injections. There was no difference between the patients given the different initial treatment protocols in age or duration of diabetes at first injection, however, those on the five and six injection loading protocols had better VA at the start (three or four more letters), so whilst there was no difference in the gain, the VA at 1 year was better.

The number of injections during the 3 years of treatment varied slightly depending on the starting loading strategy, but overall, there was no statistically significant difference in the final number of injections regardless of which loading protocol was used. Of those who were lost to follow-up 42% had an injection at their last visit.

Additional focal laser was administered to 5.3% in the 1st year, 10.3% in the 2nd and 13.4% in the 3rd year. An additional 259 patients, not included in our analysis as we only included those given aflibercept alone, had been started on aflibercept but subsequently switched to Ozurdex (241) or Iluvien (18).

Using survival modelling with loss to follow-up as the event of interest we found that those with better baseline VA are more likely to continue to attend (70 or more EDTRS letter compared to <50, Hazard ratio 0.68 (0.55–0.84) and those in the oldest age group are least likely to attend (>75 compared to <55 years, Hazard ratio 1.28 (1.02–1.61).

**Fig. 2:**
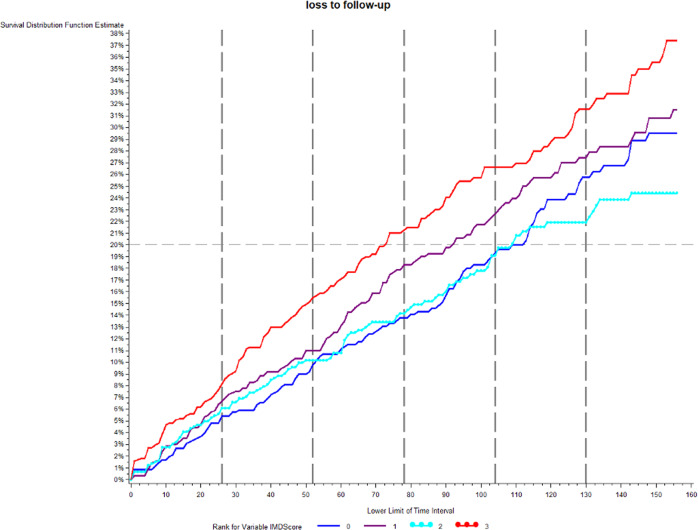
Percentage lost to follow-up over time in months in relation to the index of multiple deprivation score (IMD). The IMD has been grouped into four bands with least deprived being 0 and most 3 for the purpose of this analysis.

The IMD was correlated with age at first injection and VA at first injection. The most deprived were younger (*p* = 0.0017) and had worse VA at first injection (*p* = 0.0001). The number of injections was not related to deprivation (*p* = 0.91). There was no correlation between IMD and change in VA Hence the most deprived started from a lower baseline VA and had worse vision at 1 year (*p* = 0.0001) Those in the most deprived group were more likely to be lost to follow-up, 15% of the most deprived versus 10% in the less deprived groups by end of 1 year (Log rank test *p* = 0.0010 (Fig. 2).

## Discussion

Our study found that mean VA at which treatment is started has risen from 51 letters to 62 (Median 65) since the last RWE in DME data were published from the UK [6]. This is in parallel with a starting VA rise seen in clinical trials, such as 57 in RISE and RIDE and 60 in VIVID and VISTA [3, 4].

The DRCR.net has found that eyes with lower VA at baseline were more likely to achieve larger gains in VA but less likely to reach near-normal VA with only 17% of eyes with 25–50 letters reaching 72 letters or better with treatment [9]. Good starting VA is therefore crucial for good outcomes, however, recent data support that for those >72 ETDRS letters delaying treatment over 2 years may not actually lead to a worse outcome [10].

We found a five mean letter gain after a relatively low number of injections compared to clinical trials in the first year (mean no = 6). However, this was nearly double that of a previous UK RWE report in which the mean number of injections was 3.3 [6]. This was however reported using ranibizumab before aflibercept was available. A five letter mean gain is about half of that observed in VIVID and VISTA. The baseline VA of our patient cohort was marginally higher at 62 (Median 65) compared to the baseline VA of 60 in the pivotal trials but the mean VA achieved was a lower at 67 (Median 71) vs 70 suggesting there is room for improvement if more injections were given. Indeed, we found that the likelihood of gaining ten letters increased with each additional injection. The VA in our study was tested with habitual correction rather than refraction each time so was likely to have underestimated VA. In a UK prospective data collection study, which used refracted VA, the baseline VA was 69.5 letters [11].

Retention of DME patients is problematic in most clinical settings. In one RWE reporting on 117 patients, a gain of 4.8 and 9.6 letters were seen over 1 and 2 years, starting at 60 letters, with 5.5 injections at 1 year and a total of 8.7 at 2, however, only 31 patients had data at 2 years [12]. In the APOLLON study, a prospective 1-year follow-up RWE study in France 147 patients were included in the analysis but the cohort started at 402 (77 treatment naive). The baseline VA was 62.7 letters increasing by +7.8 letters after a mean of 7.6 injections at 1 year [13].

The Fight Retinal Blindness registry reported visual gains of +1.4 letters for 217 eyes receiving aflibercept with 70 or better VA at baseline and +10.6 with those <65, using a mean of 8 aflibercept injections at 12 months, 21% were lost to follow-up, compared to 10% at 1 year in our study [14].

A larger multicentre study from Japan of 2049 eyes measured a mean gain of 2.2 letters using a mixture of treatments. For patients just given anti-VEGF injections it was +4.5 but only 4.3 injections were given over 2 years. This study was unable to account for patients who did not have data at 2 years [15].

RWE reported from America from the Vestrum Health Retina Database had 15,608 DME patient eyes in the analysis. In the 12-month cohort, of 1379 eyes initially treated with aflibercept, the mean 12-month improvement was +5.5 letters (95% confidence interval [CI] +4.5 to +6.6 letters, *P* < 0.001) from a baseline of 57.9, after 7.5 injections on average [16].

Recommendations by a UK expert panel on the use of aflibercept in DME, published in a 2020 report that, “most panel members use six initial 4 weekly doses, as in Protocol T, rather than five initial monthly doses as recommended in the Summary of product characteristics (SmPC)” [17]. Our data show that only a small percentage of patients received 5 or 6 four weekly injections, either because this was not possible or not felt necessary by the clinician or patients.

The use of additional focal laser rose from 5.3% at the end of the first year to 13.4% at 3 years which was a lot lower than in the DRCR.net protocol T study in which laser varied from 30–40% [5].

Loss to follow-up is a weakness of our study for assessing VA outcomes, especially by 3 years, however, loss to follow-up rates is an important outcome in itself as this is a very important issue for the effectiveness of the treatment. This is a large study from 21 centres showing this is a widespread problem. Some missing data were due to incomplete EMR system use in some centres. Missing annual data were partly due to patients not attending regularly or not needing to be seen within the 8-week anniversary window. We found that attrition was not random with an increased rate for those starting with lower vision and being of older age also patients were more likely to attend if they were actively having injections. By 3 years a proportion of patients may have been discharged back to diabetic eye screening if they were stable. Death was recorded in 119 (4%) but this may have been an underestimate due to variation in how the EMR links to other records. Full data on comorbidities were not available, and these, especially strokes and heart attacks, could considerably change attendance and treatment frequency.

We found that those in the most deprived areas were also more likely to be lost to follow-up, be younger and have worse vision at the first injection. On the positive side, the number of injections and the change in VA were not related to deprivation suggesting that once aflibercept treatment was started, patient care was not compromised as a consequence of deprivation status. A previous large study of 79,775 patients in the UK also found an association between later presentation with worse VA for patients with diabetic retinopathy who were from more deprived areas [8]. Our data show that part of the differences in VA outcomes amongst the centres included in this study can be explained by the variations in the age profile of patients, their initial VA and the deprivation levels. Further work is needed to understand reasons for non-adherence to visit schedules and to find solutions [18, 19].

## Summary

### What was known before

- Randomised clinical trials of aflibercept for DME show it can improve vision. In clinical practice, the same results may not be achieved

### What this study adds

- There was variation between centres for Index of multiple deprivation, age and baseline visual acuity. These factors affected the visual acuity outcomes. The provision of Initial injection protocols of 3–6 monthly injections varied greatly between centres but did not affect the mean Visual acuity at 1 year.
